# Author Correction: Chemogenetic generation of hydrogen peroxide in the heart induces severe cardiac dysfunction

**DOI:** 10.1038/s41467-020-20668-1

**Published:** 2021-01-07

**Authors:** Benjamin Steinhorn, Andrea Sorrentino, Sachin Badole, Yulia Bogdanova, Vsevolod Belousov, Thomas Michel

**Affiliations:** 1 Department of Medicine, Division of Cardiology, Brigham and Women’s Hospital, Harvard Medical School, 75 Francis Street, Boston, MA 02115 USA; 2 Shemyakin-Ovchinnikov Institute of Bioorganic Chemistry, Russian Academy of Sciences, GSP-7, Ulitsa Miklukho-Maklaya, 16/10, Moscow, 117997 Russia; 3 Pirogov Russian National Research Medical University, Moscow, 117997 Russia; 4 Institute for Cardiovascular Physiology, Georg August University Göttingen, D-37075 Göttingen, Germany

**Keywords:** Animal disease models, Stress signalling, Heart failure

Correction to: *Nature Communications* 10.1038/s41467-018-06533-2, published online 2 October 2018.

The original version of the Supplementary Information associated with this article included an error in the Supplementary Data 1 file, where the promoter sequence in the AAV-cTnT-HyPer-DAAO-NES construct was incorrect. Supplementary Data 1 has now been corrected in the HTML version of the article; the correct version of Supplementary Data 1 can be found as Supplementary Information associated with this correction.

## Supplementary information

Supplementary Data 1

