# Supplementary material for: Chemogenetic generation of hydrogen peroxide in the heart induces severe cardiac dysfunction
Source: Nat Commun. 2018 Oct 2;9:4044. doi: 10.1038/s41467-018-06533-2 (PMC6168530; doi:10.1038/s41467-018-06533-2)
Supplement: Supplementary file 1 — Supplementary Information [file 41467_2018_6533_MOESM1_ESM.docx]

Supplementary Information:

Supplementary Figure 1: Expression of HyPer-DAAO in rats following injection with AAV9 A) immunoblots of cardiac lysates from animals infected with AAV9 carrying HyPer-DAAO-NES (DAAO) or HyPer3 (Ctrl) and treated for 4 weeks with oral D-alanine. B) Distribution of relative expression of HyPer and HyPer-DAAO from data in panel b. Data are represented as mean ± standard error.

Supplementary Figure 2: Supplementary ratiometric images. Additional representative ratiometric fluorescent images of cardiac myocytes isolated from rats infected with AAV9 carrying HyPer-DAAO-NES and stimulated with 5 mM D- or L-alanine for 45 minutes. The colorbar represents color-mapping of HyPer ratios, with a higher ratio indicating increased oxidation of the probe as described in the methods section.

Supplementary Figure 3: Control distributions for qPCR experiments. A and B) Distributions for cardiac myocytes isolated from rats infected with AAV9 carrying HyPer-DAAO-NES and treated with 10 mM L-alanine for 120 minutes. These data served as the controls to which the data in Figure 1D and 1E were normalized. C and D) Distributions of qPCR data from rats infected with control virus and treated with oral D-alanine for 4 weeks. These data served as the controls to which the data in Figure 4A and 4B were normalized. Data are represented as mean ± standard error.

Supplementary Figure 4: Effects of L-ala vs. D-ala treatment of rats expressing DAAO. Echocardiogram-determined ejection fraction of rats infected with AAV9-DAAO and fed L- or D-alanine in their drinking water for 5 weeks. ** indicates p<0.01 by t-test. Data are represented as mean ± standard error.

Supplementary Figure 5: Biochemical profile of rats expressing DAAO vs. control virus. Body weights and plasma basic biochemical parameters from control or DAAO animals fed D-alanine for 4-5 weeks.

Supplementary Figure 6: Full Western blot membranes for phospholamban: Complete membranes for phospho-serine 16 (top), phospho-threonine 17 (middle) and total (bottom) phospholamban for the cropped images that appear in Figure 4C.

Supplementary Figure 7: Full Western blot membranes for cTnT and vinculin: Complete membranes for cardiac troponin T and vinculin for the cropped images that appear in Figure 4.

Supplementary Figure 8: Full Western blot membranes for galectin-3 and vinculin: Complete membranes for galectin-3 and vinculin for the cropped images that appear in Figure 6E.

Supplementary Figure 9: Full Western blot membrane of tissue lysates from animals injected with control HyPer3 virus or HyPer-DAAO-NES virus probed for YFP and GAPDH. Complete membrane of tissue lysates from animals injected with AAV9 carrying cDNA for HyPer3 (left half of membrane) or HyPer-DAAO-NES (right half of membrane). The left image shows the membrane probed for YFP and the right image shows the same membrane probed for GAPDH. The cropped right membrane appears in Figure 1. Note that because the membrane had been blotted for YFP prior to GAPDH, bands for HyPer and HyPer-DAAO-NES remain present. The cropped right membrane appears in Figure 1.

Supplementary Figure 10: Full Western blot membrane of cardiac lysates probed for YFP and GAPDH. Complete membrane of cardiac lysates from animals injected with AAV9 carrying cDNA for HyPer3 (left 5 lanes) or HyPer-DAAO-NES (right 7 lanes) probed for YFP (left) and GAPDH (right). Note that the membrane had been blotted for YFP prior to GAPDH, and bands for HyPer and HyPer-DAAO-NES therefore remain present. The cropped membranes are displayed and quantified in Supplementary Figure 1.

**Supplemental Methods:**

| **Transcript** | **Forward Primer (5’->3’)** | **Reverse Primer (5’->3’)** |
| --- | --- | --- |
| *Hprt* | TTCCTCCTCAGACCGCTTTTC | ATCACTAATCACGACGCTGGG |
| *Hmox1* | CAACATTGAGCTGTTTGAGGAGC | GAGCGGTGTCTGGGATGAAC |
| *Nqo1* | TCCAGAAACGACATCACAGGG | GGCACCCCAAACCAATACAA |
| *Sxn1* | AGCAACCTCCTGATACCCCA | GAACGGAACCCCCTCATTCT |
| *Txnrd1* | CTCTCTTTATCCTCAGTGTGCTT | CCCGCCGCCCTATGA |
| *Il1b* | CGACAAAATCCCTGTGGCCT | TGTTTGGGATCCACACTCTCC |
| *Tnfa* | ATCGGTCCCAACAAGGAGGA | CCGCTTGGTGGTTTGCTACG |
| *Icam1* | AGCATTTACCCCTCACCCAC | AGTTCACAGTCTTGCCCCTC |
| *Nos2* | TGGTGAGGGGACTGGACTTTT | TTCTCCGTGGGGCTTGTAGT |
| *Nppa* | TGAGCGAGCAGACCGATGAA | GAGACGGGTTGACTTCCCCA |
| *Nppb* | ACAAGAGAGAGCAGGACACCA | TAAGGAAAAGCAGGAGCAGAATCA |
| *Myh6* | CTGAAGAGGCGGAGGAACAGG | TATTGTGGGATAGCAACAGCGA |
| *Myh7* | CTCCCAGAACACCAGCCTC | CCGCCTCCTCCACCTCT |
| *Col1a1* | AAGAACGGAGATGATGGGGAA | ATCCAAACCACTGAAACCTCT |
| *Col3a1* | GAATGGTGGCTTTCAGTTCAG | GCAGTGGTATGTAATGTTCTGGG |
| *Tgfb1* | CTGAACCAAGGAGACGGAATACA | AGGAGCAGGAAGGGTCGGT |
| *Mmp2* | TGCCCAGAGACTGCTATGTCC | GCACACCACACCTTGCCATC |
| *Prdx1* | CCCTCTTGACTTTACTTTTGTGTG | ATCCTCCTTGTTTCTTGGGTGT |
| *Prdx2* | TGGACTTCACTTTTGTTTGCCC | CCTCCTTCCGTGGGGTATTG |
| *Prdx3* | GTGCTTTTCTTCTACCCTTTGGA | CCACCATTCTTTCTTGGCGTG |
| *Prdx5* | GGCGTAGTAAAGGCACTGAA | AGTTGTGAGAGGATGTTGGGG |
| *Gpx1* | CCGGGACTACACCGAAATGA | ACCATTCACCTCGCACTTCT |
| *Gpx3* | GCTTCCCGTTCCAAATGAGC | GAAGGAGGCTGGTGGCATAG |
| *Txn1* | AGTAGACGTGGATGACTGCC | CACCAGAGAACTCCCCAACC |
| *Txn2* | AGAAGCCACCAGCACTTCAG | GGAGCACACAGTAGGAGGTG |

Supplementary Table 1: qPCR Primers: Gene-specific primers for qPCR amplification of mRNA transcripts.
