# Supplementary material for: Chemogenetic generation of hydrogen peroxide in the heart induces severe cardiac dysfunction
Source: Nat Commun. 2018 Oct 2;9:4044. doi: 10.1038/s41467-018-06533-2 (PMC6168530; doi:10.1038/s41467-018-06533-2)
Supplement: Supplementary file 4 — Description of Additional Supplementary Files [file 41467_2018_6533_MOESM4_ESM.docx]

**Title:** Supplementary Dataset 1

**Description:** Nucleotide Sequences of Plasmids
